# Exploring Factors Influencing Pre-Service Teachers’ Intention to Use GenAI for Instructional Design: A Grounded Theory Study

**DOI:** 10.3390/bs15091169

**Published:** 2025-08-28

**Authors:** Ruixin Wu, Xin Wang, Yong Nie, Peipei Lv, Xiande Luo

**Affiliations:** Faculty of Education, Shaanxi Normal University, Xi’an 710062, China

**Keywords:** pre-service teachers, GenAI, instructional design, usage intention, grounded theory

## Abstract

Generative artificial intelligence (GenAI) is advancing rapidly and is increasingly integrated into educational settings. How to effectively leverage GenAI to support instructional design has thus become a critical issue in teacher education. While existing studies have validated the technical potential and functional value of GenAI in instructional design, there remains a notable gap in qualitative investigations into pre-service teachers’ subjective willingness to adopt GenAI and its underlying influencing factors. To address this gap, this present study employed grounded theory to explore the factors that shape pre-service teachers’ intention to use GenAI for instructional design. Semi-structured interviews were conducted with 23 pre-service teachers from Shaanxi Normal University, and the data were analyzed through open coding, axial coding, and selective coding. A theoretical model comprising four major dimensions was developed as follows: (1) technical factors (relative advantage and ease of use), (2) environmental factors (social impact, opinion leader, and facilitating conditions), (3) usage characteristics (purpose of use and method of use), and (4) psychological factors (trust, perceived risk, and a professional self-concept). The findings reveal that pre-service teachers’ intention to use GenAI is not shaped by a single factor but is instead the result of dynamic and interrelated interactions among the four dimensions. This study extends current technology acceptance theories and offers practical insights for the effective integration and promotion of GenAI in instructional design.

## 1. Introduction

As one of the most advanced technologies in the contemporary digital landscape, generative artificial intelligence (GenAI) has received widespread attention in the international community. With continuous breakthroughs in algorithms and a dramatic increase in computing power, GenAI is developing at an unprecedented rate and is gradually becoming an important force that changes the ecological pattern of various industries (Adiguzel et al., 2023). GenAI shows great potential and broad application prospects in image generation, natural language processing, audio synthesis, and text generation, etc., and is able to deeply mine data information and create new content accordingly (Farrokhnia et al., 2024; Orhan et al., 2024). These features have brought about significant changes in education and teaching (Mao et al., 2023; C. Wang et al., 2024). In particular, GenAI’s features, such as question-and-answer, deep semantic understanding, and image recognition, can assist teachers in teaching and reduce the burden of lesson planning (Chiu, 2021; Evmenova et al., 2024; Stefaniak & Moore, 2024; Trust et al., 2023). Therefore, both in-service and pre-service teachers, in addition to the basic qualities of a teacher, need to have the ability and awareness of working collaboratively with GenAI (Bienkowski et al., 2012). Instructional design—which is widely regarded as a blueprint that guides the organization and execution of teaching activities—plays a central role in achieving effective learning outcomes. In the age of GenAI, instructional design is encountering new possibilities and challenges (Krushinskaia et al., 2024; Kumar et al., 2024). Recent studies have shown that GenAI can significantly enhance the efficiency and quality of instructional design by offering intelligent support in areas such as goal formulation, activity planning, and instructional resource integration (X. Wang et al., 2024a). Accordingly, a critical and timely issue in teacher education is how to support and prepare pre-service teachers to effectively leverage GenAI in instructional design practices.

Although existing research has explored the application potential and effectiveness of pre-service teachers’ methods of use of GenAI to assist in instructional design, for example, Ch’ng (2023) examined the role of GenAI in the instructional design process and how the Human–Computer Interaction (HCI) approach—which involves designing user-friendly and effective interactive systems through understanding users, tasks, and environments—improves the individual learning experience. Parsons and Curry (2023) examined the level of competence of ChatGPT in helping graduate students complete instructional design assignments. In addition, existing studies have acknowledged that usage intention is a critical variable in influencing technology adoption behavior (García de Blanes Sebastián et al., 2024). This is particularly salient in the context of emerging technologies such as GenAI. For example, researchers have applied grounded theory to investigate the usage intentions and influencing factors of Chinese university students regarding ChatGPT (Liu & Zhang, 2024). However, based on an analysis of the existing literature, this study identifies four key research gaps that remain unaddressed.

First, the current research primarily focuses on the general applications of GenAI (Open Innovation Team & Department for Education, 2024), while overlooking the contextual characteristics and practical demands of instructional design as a specialized domain. Instructional design is characterized by a high degree of professionalism and goal orientation (Tessmer & Richey, 1997), which differentiates it significantly from other general application scenarios. Nevertheless, existing studies have yet to conduct in-depth analyses that are specific to this domain.

Second, although prior research has explored the usage intentions of teachers and university students regarding generative AI (Lu et al., 2024; Deng et al., 2025; Wu et al., 2025), there is a notable lack of focus on pre-service teachers as a distinct group. Although pre-service teachers are future educators, they differ markedly from in-service teachers in several dimensions, such as teaching experience, technological adaptability, and self-efficacy (Edwards & Nuttall, 2016; Saltan & Arslan, 2017). Specifically, pre-service teachers generally lack real classroom teaching experience and are more likely to be affected by factors such as technology anxiety and uncertainty about their capabilities (K. Wang et al., 2024). Therefore, these factors may significantly influence pre-service teachers’ willingness to adopt new technologies, highlighting the necessity of conducting in-depth investigations that are specifically focused on this group to uncover their unique cognitive patterns and technology adoption behaviors.

Third, existing research has primarily evaluated the technical feasibility and performance of GenAI in instructional design (Chai et al., 2025). For instance, one study conducted a SWOT analysis to examine the strengths and weaknesses of using ChatGPT 3.5 for course design (Choi et al., 2024). Other research has assessed GenAI’s performance in instructional design contexts, finding that while it shows great potential in tasks such as curriculum content development, it still faces notable challenges in terms of information accuracy (Nguyen, 2022). Overall, the current literature places greater emphasis on the functional effectiveness of GenAI applications, while relatively neglecting pre-service teachers’ subjective willingness and attitudinal expressions as actual users of the technology. A variety of influencing factors—both objective and subjective—such as teachers’ perceptions, perceived values, and perceived risks regarding GenAI-assisted instructional design, may have a substantial impact on their technology adoption behavior. However, these factors have not yet been adequately explored.

Fourth, although several quantitative studies have examined the influencing factors of pre-service teachers’ intention to use GenAI, most have relied on the technology acceptance model (TAM) or its extended frameworks. Nonetheless, these studies exhibit several limitations. For example, Acquah et al. (2024) conducted a questionnaire survey with 783 pre-service teachers, employing the extended Unified Theory of Acceptance and Use of Technology (UTAUT2) to investigate how variables, such as performance expectancy, effort expectancy, habit, hedonic motivation, social influence, and facilitating conditions, affect their willingness to integrate AI into instructional planning (Acquah et al., 2024). However, quantitative studies are inherently limited by their reliance on pre-defined variables and structured questionnaire measures, which restrict their explanatory power and depth. On the one hand, pre-established models may overlook the contextual factors that are specific to instructional design and, thus, fail to fully capture teachers’ underlying intentions. On the other hand, self-reported questionnaire data are often inadequate for capturing nuanced shifts in teachers’ perceptions of GenAI. As a recent review study has noted, existing research on teachers’ adoption of GenAI is predominantly quantitative, with a noticeable lack of qualitative investigations—especially those focused on the subjective experiences of pre-service teachers (Xue et al., 2025). Therefore, relying solely on quantitative instruments such as questionnaires is insufficient to comprehensively reveal the complex mechanisms underlying pre-service teachers’ intention to use GenAI for instructional design. It is necessary to incorporate qualitative approaches so as to gain deeper insights.

In summary, although previous studies have confirmed the significant application potential of generative artificial intelligence (GenAI) in supporting instructional design and have emphasized the critical role of usage intention in determining its effectiveness, the current body of literature still lacks a systematic exploration from the subjective perspective of pre-service teachers. In particular, the key issue of “pre-service teachers’ intention to use GenAI for instructional design and its influencing factors” remains insufficiently addressed. Against this backdrop, this present study adopts a user-centered approach, focusing specifically on pre-service teachers as a key target group, and aims to systematically analyze their intention to use GenAI in instructional design, along with the influencing factors. This research seeks to fill the current gap in understanding individual-level behavior in the context of educational technology adoption. Specifically, this study addresses the following research questions: (1) What factors influence pre-service teachers’ intention to use GenAI in instructional design? (2) How do these factors interact to shape their usage intention? By answering these questions, this study aims not only to deepen the understanding of GenAI adoption behavior among pre-service teachers but also to provide both theoretical contributions and practical insights for optimizing teacher education practices and enhancing the educational design of GenAI tools.

## 2. Literature Review

### 2.1. GenAI-Supported Instructional Design

GenAI is lauded for its powerful natural language processing, code generation, and text generation capabilities, making its applications in personalized learning (Hsia et al., 2023), interactive learning (Tsang, 2023), foreign language study (Mohamed, 2023), programming learning (Jing et al., 2024), test question generation (Klang et al., 2023), paper writing (G. Hu, 2023), and evaluation (Jauhiainen & Garagorry Guerra, 2024). In response to its growing influence, the United Nations Educational, Scientific, and Cultural Organization (UNESCO) issued the “Guidelines for Action in Higher Education in the GenAI Era” to promote the standardized and ethical integration of GenAI technologies in educational contexts.

GenAI supports teachers in creating scenarios, designing real-life examples, and accessing resource support to enhance the connection between course content and the real world (P. Wang et al., 2025). It is capable of generating inspiring and creative texts that are tailored to teachers’ personalized instructional needs, thereby serving as both a source of instructional material and a catalyst for broadening pedagogical thinking (M. Wang et al., 2024). It has been found that the ‘intelligent teacher’, which is a GenAI, can set tasks and provide related materials according to specific topics, inspire teachers’ ideas and provide multiple alternatives in the process of lesson preparation, shorten the time for teachers to collect resources, and improve the efficiency of lesson preparation and the quality of lessons (Song & Lin, 2023; T. Wang, 2023). For teachers, the ability to master diverse instructional design tools and acquire corresponding technical competencies remains essential (Hodell, 2015; Rothwell & Kazanas, 2011). However, while GenAI has great potential to help instructors accomplish tasks such as generating course materials, providing advice, and acting as a virtual tutor for students by answering questions and facilitating collaboration, it also poses a number of challenges. These challenges include the generation of inaccurate or fabricated content, the potential weakening of learners’ independent thinking skills (Farrokhnia et al., 2024), and concerns related to academic integrity and ethical risks (Choi et al., 2024). Meanwhile, in practical applications, there are some teachers who have concerns about the current state of GenAI in instructional design for educational purposes (Zawacki-Richter et al., 2020). Some teachers have attempted to use GenAI to assist in instructional design a few times and then subsequently abandoned it as a regular instructional design tool. The underlying reasons for this phenomenon are multifaceted and warrant further investigation. On the one hand, some teachers reported that they feel clueless when operating GenAI tools (Budhathoki et al., 2024; Stefaniak & Moore, 2024). They often struggle with how to formulate effective prompts, and the complexity of the interface requires significant time and effort to navigate, making it difficult to achieve proficient use. On the other hand, although some teachers were able to use GenAI to generate some teaching content initially, the generated outputs often deviated substantially from authentic instructional requirements (Choi et al., 2024). The need for substantial post-editing and adaptation to align with classroom contexts discouraged teachers, who perceived that GenAI failed to deliver on its promise of efficiency and convenience. In addition, some teachers were concerned about the possible copyright issues of the teaching designs generated by GenAI, or that over-reliance on GenAI would diminish their teaching ability and creativity (Allen et al., 2024; Bakla, 2023; Eke, 2023). Future educators must be equipped to integrate GenAI effectively into instructional practice. Therefore, pre-service teachers should deeply understand the operation mechanism of GenAI, accurately identify the advantages and disadvantages of AI-generated content, and skillfully use GenAI tools to optimize and improve the teaching process to meet the new trends and new needs of educational development.

### 2.2. Study on Continued Usage Intention of New Technology

Emerging technologies can exert a significant impact on the field of education, and there are a number of well-established theoretical frameworks for examining individuals’ acceptance and willingness to adopt technology. As one representative example, GenAI is a type of emerging technology, and these theoretical frameworks can be applied to examine pre-service teachers’ intentions to use GenAI. For example, Yuriev et al. (2020) investigated the influencing factors of Chinese college students’ intentions to adopt GenAI based on the Theory of Planned Behavior (TPB), a widely recognized psychosocial model that has demonstrated robust predictive power in explaining human behavioral intentions. Hu et al. examined the factors influencing Chinese pre-service teachers’ behavioral intention and future use of GenAI for instructional purposes based on the Unified Theory of Acceptance and Use of Technology 2 (UTAUT2), and found that effort expectancy, social influence, hedonic motivation, and habit positively influenced pre-service teachers’ behavioral intention to use GenAI in their future teaching and learning. Perceived risk had a negative effect on their continued use intention, while performance expectancy, facilitating conditions, and price value showed no significant impact (L. Hu et al., 2025).

Instructional design serves as a critical foundation for successful teaching, directly influencing the quality of instruction and students’ learning outcomes. In the context of the rapid advancement of GenAI, understanding the factors that influence teachers’ use of GenAI to support instructional design has become a new focus of research. K. Wang et al. (2024) applied the Unified Theory of Acceptance and Use of Technology (UTAUT) to integrate multiple variables and found that GenAI anxiety, social influence, and performance expectancy significantly predicted pre-service teachers’ intentions to engage in GenAI-assisted instructional design, while effort expectancy and facilitating conditions showed no statistically significant relationship with their behavioral intentions (Asrori & Setyaningsih, 2025). Furthermore, they investigated the acceptance of using GenAI for lesson planning among high school geography teachers based on the technology acceptance model (TAM) and found that these teachers generally exhibited a positive attitude toward using GenAI, although they also expressed certain concerns about its future use. Other studies have employed the UTAUT2 model and structural equation modeling to reveal that performance expectancy is the only significant factor influencing pre-service teachers’ use of GenAI to support various instructional tasks, including instructional design, concept development, and problem solving (Wijaya et al., 2024).

To summarize, existing research has confirmed that GenAI has great potential to provide effective guidance in assisting teachers with instructional design, generating course materials, stimulating instructional creativity, and enhancing design efficiency. However, several challenges remain regarding its integration into educational contexts. Meanwhile, most quantitative studies in the field of education have been conducted to explore pre-service teachers’ usage intentions and technical factors. Although such studies reveal macro-level correlations and trends, they often fail to capture the complex, nuanced, and context-dependent influences on pre-service teachers’ use of GenAI for instructional design. Grounded theory begins with the phenomenon under investigation (Miles, 1994). By continuously comparing and analyzing rich qualitative data, grounded theory enables an in-depth exploration of pre-service teachers’ authentic experiences, perceptions, and the contextual factors in which they are embedded. This approach facilitates the development of a comprehensive and contextually grounded theoretical framework to understand the factors influencing pre-service teachers’ use of GenAI-assisted instructional design. Therefore, this study adopts grounded theory as its methodological foundation to explore pre-service teachers’ intention to use GenAI for instructional design and to identify the factors that influence this intention.

## 3. Methodology

### 3.1. Research Method

This study aims to explore pre-service teachers’ willingness to use generative artificial intelligence (GenAI) to assist in instructional design, as well as the factors influencing this willingness. A qualitative research design was adopted, combining semi-structured interviews and grounded theory for data collection and analysis.

First, this study employed a semi-structured interview approach to collect first-hand data (DeJonckheere & Vaughn, 2019). Compared to structured questionnaires, semi-structured interviews offer greater flexibility and openness, which enables researchers to gain in-depth insights into participants’ authentic perspectives, individual experiences, and underlying motivations. When encountering emerging technologies such as GenAI, pre-service teachers may exhibit diverse usage intentions. Semi-structured interviews allow researchers to guide discussions around core themes while still providing space for participants to express themselves freely, thereby effectively capturing their subjective experiences and practical challenges. This study adopts the grounded theory approach proposed by (Glaser & Strauss, 1967). During the data analysis process, grounded theory is an important theoretical construct research method in qualitative research, which is based on existing theories and combined with practical observation and generalization to penetrate into the specific context behind the theory and analyze the respondents’ understanding, awareness, and intention (Chen, 1999). Compared with traditional qualitative methods, grounded theory underscores a systematic and rigorous research process. Researchers are required to follow a sequence of structured steps to ensure the scientific reliability of their analysis, including systematic data collection, open and axial coding, in-depth data interpretation, theoretical category construction, and repeated testing for theoretical saturation and cross-validation to ensure the credibility and validity of the findings (Stough & Lee, 2021). This method enables researchers to derive new theories or refine existing ones from complex empirical data, thereby uncovering the deeper logic and influencing factors underlying a given phenomenon (Chun Tie et al., 2019). Grounded theory is widely applied in educational research and is particularly suitable for investigating the psychological states and behavioral intentions of teachers and students when engaging with emerging technologies (Stough & Lee, 2021). Grounded theory emphasizes understanding behavioral motivations from the participants’ perspective, which enables the researcher to uncover the psychological dynamics that pre-service teachers undergo when facing GenAI. As such, it offers a deeper explanation of their technology adoption behavior.

### 3.2. Data Collection

Grounded theory research methods generally obtain primary data in the form of interviews (Charmaz, 2006). Initially, the interview protocol was revised and refined based on feedback gathered during a pilot interview phase. During the pre-interview process, five pre-service teachers were randomly selected to introduce their personal feelings of using the pre-interview outline as well as their perceptions of its clarity and appropriateness. The formal interview outline was formed with responses and suggestions from the pre-interview participants.

The interviews centered on the following six questions (see Table 1):

This study focuses on pre-service teachers as the primary research participants, based on two main considerations. First, pre-service teachers’ instructional beliefs are still in the process of formation and development, making their attitudes and willingness toward emerging technologies such as GenAI more malleable (Kohnke et al., 2025; K. Wang et al., 2024). Compared to experienced in-service teachers, pre-service teachers are more susceptible to the influence of teaching practicums and external environments during the technology adoption process, which highlights the need for a deeper investigation from the perspective of factors influencing usage intention. Second, the current teacher education system is placing increasing emphasis on integrating AI literacy into pre-service teacher training (Siiman, 2024). Therefore, understanding pre-service teachers’ willingness to use GenAI for instructional design is of practical significance for optimizing teacher education curricula.

All pre-service teacher participants in this study were from Shaanxi Normal University, which is a key university that is included in the “211 Project” and is directly administered by the Ministry of Education. The university enjoys a strong social influence in the field of teacher education (X. Wang et al., 2024b), and its pre-service teachers are highly representative in terms of both technological exposure and instructional design competence. In recent curriculum reforms, the university has actively integrated GenAI technologies to support teaching practices (Shaanxi Normal University, 2025), providing participants with authentic contexts and experiential foundations for the use of GenAI. This background facilitated the collection of empirically grounded and practically relevant interview data. The screening criteria for the interviewees were as follows: (1) having used GenAI to assist in instructional design; (2) possessing their own understanding and perspectives of GenAI; and (3) demonstrating strong verbal communication skills and the ability to express ideas authentically. A total of 23 participants were ultimately selected, of which 20 interview transcripts were used for open coding, while the remaining three were retained for theoretical saturation testing. Table 2 presents the detailed demographic and academic information of the participants. Prior to the interviews, participants signed an informed consent form assuring them that all data would be used exclusively for academic research and that their personal information would remain confidential.

### 3.3. Data Analysis

The data were analyzed following grounded theory principles, employing a three-stage coding process that consisted of open coding, axial coding, and selective coding using NVivo12 Pro software as an auxiliary analysis tool. During the coding process, the data were repeatedly examined and compared to extract conceptual categories, refine core categories, and construct a research model and conceptual framework that illustrated the factors that influence pre-service teachers’ intention to use GenAI-assisted instructional design based on the relationships between these categories.

#### 3.3.1. Open Coding

Open coding is the process of disaggregating and integrating source material by disorganizing and reorganizing qualitative information, extracting original statements, abstract concepts, and categorizing them (Barsalou, 2008). To ensure the consistency and reliability of the coding analysis results, the research team conducted collaborative analysis and discussion. The research team was divided into two groups: the first and third authors formed one group, and the second and fourth authors formed another. Each group independently conducted blind coding. The coding analysis results were finalized only if the consistency of the coding results between the two groups reached 90%; otherwise, the coding process was repeated until sufficient agreement was achieved. By reading, comparing, and coding the interview text data sentence by sentence, this study summarized and refined 33 initial concepts and indigenous concepts, and further abstracted 11 initial categories. Examples of the first-level codes are provided in Appendix A.

#### 3.3.2. Axial Coding

Axial coding refers to the formation of categories through a process of further organizing and integrating open codes. This process aims to identify and establish internal relationships between different categories through cluster analysis to build a more rigorous and organic classification system (Barsalou, 2008). In this study, the 11 initial categories that were identified during open coding were further condensed and refined into five main categories: technical factor, environmental factor, usage characteristics, psychological factor, and usage intention, as shown in Appendix B.

#### 3.3.3. Selective Coding

Selective coding involves a comparative analysis of the relationships among the main categories, through which a core category is extracted that integrates all other categories (Barsalou, 2008). Based on open coding and axial coding, this study identified ‘usage intention’ as the core category. The ‘storyline’ can be described as follows: pre-service teachers’ continued willingness to use GenAI-assisted instructional design is influenced by technical factors, environmental factors, usage characteristics, and psychological factors. This is shown in Table 3.

#### 3.3.4. Theoretical Saturation Test

In grounded theory, the reliability and consistency of the three levels of coding analysis are typically verified through a theoretical saturation test, which indicates saturation when no new concepts or categories emerge during the analysis of additional data (Corbin & Strauss, 1990). In this study, three previously retained interview transcripts were used for theoretical saturation testing. The results showed that the concepts appearing in these three interview texts were already encompassed by the existing codes and categories. No additional categories or conceptual relationships were discovered, thereby confirming that the constructed theoretical model had reached theoretical saturation.

## 4. Results

**Figure 1:**
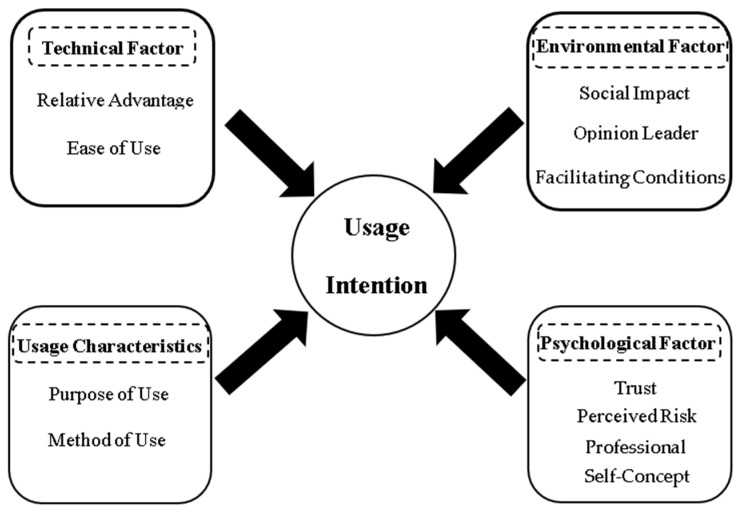
Modeling of pre-service teachers’ usage intention and influencing factors for GenAI-assisted instructional design.

Based on the analysis and coding of the interview data, this section constructs a theoretical model (see Figure 1) to explain the factors influencing pre-service teachers’ intention to use GenAI for instructional design. The model clearly demonstrates that pre-service teachers’ use of GenAI in instructional design is not the result of a single variable, but rather a comprehensive outcome influenced simultaneously by technical factors, environmental factors, usage characteristics, and psychological factors. Therefore, when exploring the factors affecting pre-service teachers’ intention to adopt GenAI-assisted instructional design, it is essential to consider all four dimensions: technical, environmental, usage-related, and psychological.

### 4.1. Pre-Service Teachers’ Usage Intention to Use GenAI to Assist Instructional Design and Influencing Factors Modeling Framework Construction

Through the coding analysis of the raw interview data and in response to the research questions, it was found that pre-service teachers’ usage intention to use GenAI-assisted instructional design can be categorized into three distinct types based on the degree of subjective willingness: positive use, neutral use, and negative use. Furthermore, this intention is influenced by four interrelated dimensions: technical factors, environmental factors, usage characteristics, and psychological factors. Therefore, this study developed a model of pre-service teachers’ usage intention and the influencing factors of GenAI-assisted instructional design based on the results of the coding and analysis. The specific information is shown in Figure 1.

### 4.2. Technical Factor

From the socio-technical perspectives, technical factors refer to the role of technology in regulating and guiding various activities within complex systems (Morgan-Thomas et al., 2020). It consists of two dimensions: relative advantage and ease of use.

The results of the study show that the technical factor is an important factor that influences pre-service teachers’ willingness to use GenAI to assist in instructional design, and there is a causal relationship with usage intention.

#### 4.2.1. Relative Advantage

Relative advantage refers to the degree to which using an innovation is perceived as being better than using its precursor (Moore & Benbasat, 1991). In this study, it refers to the practical value and positive effect of GenAI in enhancing various aspects of pre-service teachers’ instructional design, which is reflected in the efficiency, quality, resourcefulness, student interest stimulation, innovation inspiration, and personalized support. Compared with traditional instructional design, GenAI can significantly improve efficiency and optimize instructional design based on big data and advanced algorithms. For example, an interviewee (I) said, “In the past, it took several days to prepare a complete instructional design, but with GenAI, you only need to input the key elements, and the basic framework can be generated in a short period of time.” At the same time, GenAI has a powerful teaching resource library, which provides easy and quick access to subject extension materials and case studies to meet different teaching needs. As one of the interviewees (L) said, “Before, I had to switch back and forth between several websites to find suitable teaching resources. Now I can use GenAI to meet my needs.” In addition, some interviewees (E, P, and V) think GenAI’s interactive classroom design is innovative and unique, which can effectively attract students’ attention and stimulate their interest in learning. Some pre-service teachers (D, H, U, and P) think GenAI provides teachers with new teaching activity designs and unique ways of presenting knowledge, which breaks the traditional thinking of teaching and brings new teaching inspiration. Some pre-service teachers (G, K, and R) have also found that using GenAI enables them to analyze student learning data, find weak areas, and customize individualized instructional programs to meet the learning needs of different students.

#### 4.2.2. Ease of Use

Ease of use refers to the degree to which using an innovation is perceived as being difficult to use (Moore & Benbasat, 1991). In this study, it refers to the fact that GenAI provides a simple and intuitive interactive interface, allowing pre-service teachers to start quickly without complicated operation steps. The ease of use of GenAI-assisted instructional design is reflected in its simplicity and low learning curve. Since most pre-service teachers have prior experience with computer usage, several interviewees (S, F, N, and A) said that the GenAI tools are easy to operate, and they can easily realize the functions without complex operation steps. This ease of use increases their willingness to experiment with and adopt the technology. At the same time, the learning threshold of GenAI is low. As one interviewee (O) said, “I originally thought it would take a while to master GenAI, but it turns out that the learning threshold is very low. Look at the video tutorials and follow the operation a few times, and then you can skillfully use it, and the result is still very good.” Compared to traditional teaching aid software, the basic operations and core functionalities of GenAI are easier for pre-service teachers to learn and apply.

### 4.3. Environmental Factor

Environmental factor refers to the factors that can affect a person’s behavior. There are social and physical environments (Albarracín & Dai, 2024). In this study, the environmental factor comprises three key components: social influence, opinion leadership, and facilitating conditions. The results of this study demonstrate that the environmental factor is a significant determinant of usage intention, and there exists a causal relationship between environmental factors and pre-service teachers’ willingness to adopt GenAI-assisted instructional design.

#### 4.3.1. Social Influence

Social influence refers to individuals’ perceptions of influential others supporting the utilization of a new technology system (Venkatesh et al., 2003). In this study, social influence is defined as the influence of social factors on pre-service teachers’ use of GenAI to support instructional design. It comprises three dimensions: peer influence, social recognition, and online promotion. Pre-service teachers are in a group environment where educational learning and practice are integrated, and communication among peers influences their behavior of using GenAI-assisted instructional design. Peer influence manifests when individuals around them are actively experimenting with GenAI and sharing their experiences. As one interviewee (M) said, “My peer mentioned that he had recently used GenAI-assisted instructional design to design instructional sessions and extend instructional materials, and the feedback on student learning was very good, which made me want to try it too.” Mutual encouragement and experience sharing among peers can reduce pre-service teachers’ resistance to new things and motivate them to explore the application of GenAI in instructional design more actively. On the other hand, if there are misconceptions about GenAI in the peer group, the spread of negative news can discourage pre-service teachers from trying GenAI. Social recognition also influences pre-service teachers’ decision-making. As one interviewee (U) noted, parents’ positive feedback on students’ performance after using GenAI in instructional design increases their confidence in using GenAI. The confidence to use GenAI will be boosted. Online promotion plays a unique role in the information age. When large amounts of information about GenAI-assisted instructional design are disseminated through educational websites, short video platforms, public social media accounts, and other channels, they influence pre-service teachers’ usage decisions. One interviewee (C) shared, “I saw a blog post on WeChat showing a GenAI-assisted instructional plan for an elementary IT class, which included clear steps and a list of required materials.” This kind of concrete presentation, combining “case + outcomes,” reduces pre-service teachers’ unfamiliarity with the technology and enables them to perceive the practical value of GenAI intuitively.

#### 4.3.2. Opinion Leader

Opinion leader refers to those who are similar in social position to those they influence and who are perceived to be competent about the topic under discussion, and hold many diverse contacts and frequent discussions within his or her own social network (Katz, 1957). In this study, opinion leaders refer to the influence of a group of people with a certain level of prestige and social status on pre-service teachers’ use of GenAI-assisted instructional design. Opinion leaders contain two dimensions: mentor recommendations and industry trends. When mentors frequently emphasize the advantages of GenAI in instructional design during guidance sessions, it often sparks pre-service teachers’ interest in the technology. For example, one interviewee (H) noted, “My teacher demonstrated a method for designing differentiated assignments using GenAI during a group meeting. By inputting students’ varying proficiency levels, the tool automatically generated exercises with clear difficulty gradients.” Moreover, growing recognition of GenAI within the broader education sector has prompted pre-service teachers to proactively engage with the technology. One interviewee (Q) shared, “In a recent pre-service teacher training session, experts repeatedly emphasized that GenAI proficiency would become a core competency for teachers over the next five years.”

#### 4.3.3. Facilitating Conditions

Facilitating conditions refer to the degree to which an organizational and technical infrastructure exists to support the use of the system (Venkatesh et al., 2003). These components work synergistically to construct a supportive ecosystem that empowers pre-service teachers to engage in effective GenAI-assisted instructional design. Resource support provides the material foundation necessary for pre-service teachers to access and implement GenAI tools. As one interviewee (L) stated, “I can directly search for GenAI teaching cases in the university database and find instructional plans with detailed video explanations.” Professional and systematic training programs are essential for helping pre-service teachers acquire the skills and strategies needed for GenAI use. Experienced trainers not only offer hands-on guidance but also share practical techniques and insights into the latest technological trends, thereby broadening teachers’ horizons and inspiring innovation. One interviewee (T) mentioned, “In last week’s GenAI training, the instructor walked us through how to use the tool to analyze student error patterns—I now have a much clearer understanding of its functions.” Moreover, supportive policies play a pivotal role in shaping pre-service teachers’ willingness to adopt GenAI. Institutional incentives, such as integrating GenAI-related knowledge and skills into teacher education curricula, can actively encourage adoption. Several interviewees (D, E, O, K, T, and W) noted that their institutions promote the appropriate use of GenAI to assist in academic learning and instructional planning.

### 4.4. Usage Characteristics

Usage characteristics refer to the dynamic pattern of adaptation between individuals’ selection of technological functions, operational strategies, and task requirements in specific task contexts that are aimed at achieving their goals (Goodhue & Thompson, 1995). It contains two dimensions: purpose of use and method of use.

The results of this study show that features of use are important factors that influence pre-service teachers’ willingness to use GenAI to assist in instructional design, and there is a causal relationship with usage intention.

#### 4.4.1. Purpose of Use

Purpose of use refers to individuals who engage with technology to fulfill specific demands within task-based contexts (Venkatesh et al., 2003). In this study, it refers to the functional goals that pre-service teachers aim to achieve through the use of GenAI tools, which are directly aligned with the specific task demands of instructional design. This includes courseware generation as well as language editing and proofreading. In the context of fast-paced educational environments where time is a scarce resource, independently producing high-quality instructional materials can be both time-consuming and labor-intensive. One interviewee (J) shared: “In the past, it would take at least two or three days to create a polished set of slides. But with GenAI, I just input the lesson topic and key points, and within moments, a draft is generated—it not only organizes key knowledge points but also integrates relevant teaching cases and visual materials.” This greatly reduces preparation time and allows pre-service teachers to focus more on in-depth pedagogical thinking and personalized adjustments. Several interviewees (I, K, L, N, and P) also noted that GenAI helps streamline the writing of instructional texts by providing precise language expressions, saving considerable time. Moreover, for English-language teaching materials, GenAI offers grammar checking and translation enhancement, thereby supporting the development of bilingual instruction.

#### 4.4.2. Method of Use

Method of use refers to users who tend to adapt their use of technological functions based on specific task requirements in order to maximize the impact of technology on task performance (Goodhue & Thompson, 1995). In this study, the method of use refers to how GenAI is employed to support instructional design. It includes three key aspects: question formulation, screening methods, and the type of GenAI tool selected. Clear and precise questioning by pre-service teachers can guide GenAI to generate more targeted and relevant outputs. For example, one interviewee (P) mentioned, “Are there any interactive courseware templates suitable for teaching classical Chinese poetry in middle school? The template should include visual illustrations of the poetic imagery and a brief literary appreciation.” This type of specific query helps narrow the scope of GenAI’s output, enabling teachers to quickly access resources that meet their needs and thus enhancing preparation efficiency. Screening methods also play a critical role in determining whether pre-service teachers can effectively extract useful information from the large volume of GenAI-generated content. Many pre-service teachers (C, H, and J) reported filtering results based on instructional goals, students’ learning conditions, or classroom time constraints. Different types of GenAI tools offer varying advantages, and pre-service teachers tend to select specific tools according to their instructional needs and teaching contexts to achieve optimal outcomes. As one interviewee (K) shared, “I used to worry about grammatical errors in my English lesson plans. After using a text-enhancement GenAI tool, it was able to highlight awkward expressions and correct them.”

### 4.5. Psychological Factor

Psychological factor refers to the factors related to how individuals perceive and process information (Shaw & Kang, 2025). It consists of three factors: trust, perceived risk, and professional emotional association.

The results of this study show that the emotional factor is an important factor influencing usage intention, and there is a causal relationship between the emotional factor and usage intention.

#### 4.5.1. Trust

Trust refers to the willingness to rely on an exchange partner in whom one has confidence (Moorman et al., 1993). In this study, it refers to the level of trust and recognition of GenAI-assisted instructional design by pre-service teachers. In terms of trust, some pre-service teachers (C, H, J, K, L, N, and P) had a high level of trust in GenAI-assisted instructional design. For example, one interviewee stated, “It can accurately search for both subject expansion materials and cases that fit students’ interests, and the quality is very high, so I almost always use it to look for materials now.” Some other pre-service teachers (A, D, M, and W) expressed lower levels of trust in GenAI’s instructional design. For example, “The classroom interaction it recommends looks quite fancy, but in practice, students cannot participate in it at all, and it does not achieve the expected effect at all.” Although GenAI possesses a powerful resource base and supports personalized needs, it sometimes generates content that is of poor quality and is detached from practical teaching contexts, which significantly undermines users’ trust.

#### 4.5.2. Perceived Risk

Perceived risk refers to the expectation of losses associated with purchase and acts as an inhibitor to purchase behavior (Peter & Ryan, 1976). In this study, it refers to pre-service teachers’ subjective perceptions of the potential risks associated with using GenAI-assisted instructional design. It encompasses technophobia, ethical concerns, content quality, individual creativity, and the de-skilling of professional competence. These multiple dimensions of perceived risk can significantly reduce pre-service teachers’ willingness to adopt GenAI for instructional design. Such concerns stem not only from apprehensions about the technology itself, but also from reflections on their own teaching competencies and long-term professional development.

Technophobia causes resistance due to unfamiliarity with the tool. As one pre-service teacher (B) confessed, “Every time I open a GenAI tool, I feel uncomfortable because its operating logic is different from how I usually plan lessons.” Ethical concerns focus on teaching integrity and the boundaries of professional responsibility. One interviewee (Q) mentioned, “When I generate a full lesson plan using GenAI, I often wonder—if I use this directly in class and students find out I didn’t write it myself, will they question my professionalism?”. Uncertainty over content quality increases anxiety. One participant (S) noted, “I once used GenAI to generate math exercises, and two formulas were wrong. Luckily, I caught the mistake during preparation. Now, every time I use it, I have to check line by line, which is even more time-consuming than writing my own questions.” Concerns over personal creativity led teachers to be wary of over-reliance. Another participant (J) expressed, “The lesson plans generated by GenAI always feel similar. I’m worried that, over time, my own teaching designs will become repetitive and uninspired.” Fears about professional de-skilling further heighten caution. One interviewee (R) reflected, “If GenAI does everything, will my own lesson planning and problem-solving abilities deteriorate?” These career development concerns directly limit their usage frequency. The cumulative effect of these perceived risks results in pre-service teachers approaching GenAI with caution rather than trust, and in some cases, even choosing to actively avoid using it.

#### 4.5.3. Professional Self-Concept

The professional self-concept refers to an individual’s perception of their own professional role, competence, and value (Super, 1980). In this study, the term professional self-concept refers to the impact of GenAI-assisted instructional design on pre-service teachers’ professional identity and sense of professional value. It encompasses both their sense of professional identity and perceived professional value. In terms of professional identity, pre-service teachers showed complex emotional experiences when using GenAI-assisted instructional design. One interviewee (U) said, “Using GenAI to analyze the learning situation is efficient and accurate. This makes my lesson planning more focused, and I feel like I’ve kept up with the cutting edge of technological development.” This sense of efficiency and precision in lesson planning, enabled by GenAI, not only enhanced pre-service teachers’ confidence in their instructional practices but also reinforced their identification with the teaching profession.

However, some pre-service teachers also expressed concerns. An interviewee (J) said, “Sometimes I rely too much on GenAI, and my instructional design is almost always based on what it generates, with little thinking on my part. In the long run, I worry that my teaching characteristics will be eroded.” When instructional design becomes overly reliant on GenAI and lacks personal reflection and innovation, pre-service teachers may develop self-doubt and gradually lose their professional identity. Professional value perception plays a positive role in promoting pre-service teachers’ willingness to use GenAI for instructional design. A higher sense of professional value helps strengthen their intention to adopt the technology, while a lower sense of professional value may weaken their intrinsic motivation for active use. For example, some pre-service teachers believe that GenAI can support teaching and thereby enhance their professional value. One interviewee (D) mentioned, “The diverse teaching cases provided by GenAI gave me inspiration. After integrating them with my class context, students responded that the lesson was very interesting. This made me feel that using GenAI helped me better fulfill my teaching responsibilities.” However, some teachers experienced value-related anxiety due to concerns that GenAI might diminish their irreplaceability, leading to resistance. “Seeing that GenAI can quickly generate a complete lesson plan, including class questions and post-class reflections, I worry that if all these can be replaced by technology, what value do my teaching reflections and personal experiences still hold?” said another interviewee (B). These findings suggest that the impact of professional value perception on the use of GenAI is essentially a process of seeking balance between technological assistance and self-worth realization. When GenAI is perceived as a tool that amplifies one’s educational value, it facilitates adoption; when it is viewed as a threat to one’s uniqueness, it inhibits usage.

### 4.6. Usage Intention

Usage intention refers to the subjective tendency of an individual to actually use a technology, tool, or product, based on their awareness of its existence, their cognition, and attitude toward it, as well as contextual considerations (Ajzen, 1991). In this study, usage intention refers to pre-service teachers’ subjective inclination to adopt and apply GenAI during the instructional preparation phase, based on their perceptions, attitudes, and contextual considerations. It encompasses active usage intention, negative usage intention, and neutral usage intention.

With regard to usage intention, pre-service teachers may actively and deliberately integrate GenAI tools into various stages of instructional design. As one interviewee (L) stated: “GenAI has significantly reduced the time I spend on lesson preparation while enhancing overall teaching effectiveness. Now, whenever I need to design instruction, turning to GenAI has become my first instinct.” This proactive intention stems from pre-service teachers’ recognition of the supportive role of GenAI, as they believe it can enhance the efficiency and richness of instructional design, thereby encouraging them to explore the tool’s various functions. In contrast, negative usage intention is characterized by pre-service teachers’ reluctance or unwillingness to adopt GenAI-assisted instructional design. As one interviewee (M) noted: “While GenAI offers certain advantages in instructional design—such as convenient access to relevant resources—it also has limitations. For instance, the generated content is not always well-aligned with real classroom contexts. Therefore, I decide whether to use it based on the specific needs of each teaching situation.” Such a negative intention may arise from a lack of trust in the tool or concerns about the development of one’s own professional competence, leading to passive or even resistant behavior during use. Neutral usage intention is observed among pre-service teachers who show no strong preference for or against using GenAI in instructional design. Their decisions tend to depend on the specific context. As one interviewee (N) stated, “Sometimes, when I have enough time to prepare, I prefer to plan the lesson myself. But if I’m pressed for time, I’ll use GenAI to help complete part of the work. I wouldn’t say I’m particularly proactive, but I don’t reject it either.” These teachers regard GenAI as an optional tool, and whether they use it depends on their practical needs rather than a strong subjective inclination.

These situations indicate that usage intention has a direct impact on pre-service teachers’ behavior regarding the use of GenAI in instructional design. A proactive intention encourages them to actively utilize the tool, while a negative intention inhibits their engagement. In contrast, a neutral intention leads them to fluctuate between using and not using GenAI, with their actual behavior being largely influenced by external conditions.

## 5. Discussion

Based on a systematic analysis grounded in grounded theory, this study developed a theoretical model to explain pre-service teachers’ intention to use generative artificial intelligence (GenAI) to support instructional design. The model suggests that pre-service teachers’ usage intention is not driven by a single factor, but rather results from the synergistic effects of multiple variables, specifically including four dimensions: technical factors, environmental factors, usage characteristics, and psychological factors. The following sections provide an in-depth discussion of each of these four dimensions, analyzing their specific manifestations, comparing them with existing studies, and exploring their theoretical contributions. Finally, practical implications and directions for future research are proposed.

### 5.1. Interpreting Usage Intention

The findings of this study indicate that usage intention is a significant antecedent variable of users’ adoption behavior. Specifically, when pre-service teachers exhibit a higher intention to use GenAI for instructional design, they tend to use GenAI more frequently and effectively in practice. This finding is consistent with previous research on technology acceptance models. Prior studies have pointed out that usage intention is a key predictor of whether individuals will adopt emerging educational technologies, serving as a mediating variable between cognitive judgments and actual behavior (Venkatesh et al., 2003). In addition, usage intention also reflects pre-service teachers’ positive expectations regarding their teaching competence and professional identity. When they are willing to enhance teaching quality and classroom performance through technological means, GenAI is perceived as an effective tool for facilitating professional growth and instructional innovation.

Based on the findings of this study, future research could further advance in the following directions. First, it is necessary to explore whether usage intention can be considered a core variable that influences pre-service teachers’ professional development, thereby expanding its theoretical significance within teacher education research (Torsney et al., 2019). Second, longitudinal investigations could be conducted to track the dynamic evolution of pre-service teachers’ usage intention of GenAI across different learning stages or instructional tasks, aiming to uncover the underlying mechanisms and influencing factors that contribute to the formation, reinforcement, or weakening of such intention. Finally, comparative studies across different countries, regions, or disciplinary contexts could be carried out to enhance the generalizability of research findings. In summary, usage intention, as a central variable in technology adoption research, not only helps predict pre-service teachers’ technology use behavior but also reflects their attitudes and adaptive responses in the face of technological transformation.

### 5.2. Interpreting Technical Factors

The findings of this study indicate that technical factors consist of two components: relative advantage and ease of use, which together form the dimension of technical factors. Specifically, when users perceive that a new technology offers a clear relative advantage over existing solutions and is easy to use, they are more likely to accept and adopt it. This is consistent with previous research findings, where studies have shown that people are more inclined to adopt new technologies that exhibit high relative advantage and low complexity (Skoumpopoulou et al., 2018; X. Wang et al., 2024a). In addition, empirical research has also confirmed this perspective, showing that perceived ease of use and relative advantage—two key elements in the TAM and IDT models—are significant determinants of technology adoption (Ramli et al., 2017).

Interview data from this study revealed that improving the ease of use of a technology can enhance users’ perception of its advantages, thereby increasing their willingness to adopt it. This finding is consistent with the perspective of extended models of the technology acceptance model (TAM), which suggests that the combination of perceived usefulness (relative advantage) and ease of use provides a more comprehensive explanation of users’ acceptance behavior. The interaction between these two factors jointly influences users’ adoption attitudes (Venkatesh & Davis, 2000). This relationship may have emerged in the present context because pre-service teachers tend to value the tangible benefits that GenAI technologies can bring to instructional design (i.e., relative advantage). Enhancing ease of use can amplify these benefits and reduce usage barriers, thereby indirectly promoting the acceptance of GenAI technology. This offers a theoretical explanation for the mechanism of technical factors in the context of this study.

In future research, continued attention should be paid to how technical factors can be optimized to enhance pre-service teachers’ willingness to use GenAI. Specifically, improvements can be made in the following two aspects. First, the relative advantage of GenAI should be enhanced by continuously iterating and optimizing the tool so that its features better align with the practical contexts and needs of instructional design (Alkhasawneh et al., 2025; Gonsalves, 2025). Second, the ease of use of the technology should be improved by lowering the learning threshold and simplifying the user interface. Through the synergistic enhancement of these two dimensions, pre-service teachers’ adoption and application of GenAI in instructional design can be effectively promoted.

### 5.3. Interpreting Environmental Factors

The findings of this study indicate that environmental factors consist of social influence, opinion leaders, and facilitating conditions. These elements collectively shape pre-service teachers’ intention to use GenAI. Previous research supports this perspective. For instance, empirical studies have demonstrated that both social influence and facilitating conditions significantly affect employees’ willingness to adopt e-government systems. Meanwhile, the Innovation Diffusion Theory (IDT) suggests that opinion leaders play a crucial role in technology dissemination: when opinion leaders in a social system are the first to adopt and promote an innovation, the number of new adopters per unit of time increases exponentially (Henderson, 2017). Thus, a supportive social environment (e.g., encouragement and modeling from peers) and adequate external support conditions (e.g., resources and technological infrastructure) are essential to promoting pre-service teachers’ adoption of GenAI in instructional design.

The interview findings revealed that peer support and contextual environment play a significant role in promoting pre-service teachers’ intention to use GenAI. This indicates that not only are the inherent attributes of GenAI important, but also support from others and environmental influences significantly shape pre-service teachers’ willingness to adopt the technology. This finding expands the existing theoretical models by highlighting the impact of environmental factors. In this study, environmental factors proved to be indispensable, providing new contextual evidence for their significant influence on behavioral intention.

The findings on environmental factors offer valuable implications for future practice. First, teacher education programs should foster a positive atmosphere for integrating GenAI into instructional design. For instance, teacher educators can incorporate GenAI in their own instructional design practices and share successful experiences to stimulate pre-service teachers’ willingness to use the technology (Chan & Lee, 2023). Second, efforts should be made to improve the facilitating conditions for GenAI adoption, including providing comprehensive training support and technical assistance, thereby reducing pre-service teachers’ concerns when using GenAI (Bae et al., 2024). Future research may further explore the mechanisms of environmental factors across different cultural and contextual settings, in order to develop more targeted strategies for promoting GenAI use.

### 5.4. Interpreting Usage Characteristics

This study demonstrates that usage characteristics consist of two dimensions: purpose of use and method of use. These characteristics reflect why and how pre-service teachers utilize GenAI for instructional design, which directly influences the effectiveness of use and user experience. Prior research has indicated that users’ willingness to adopt a technology and their satisfaction with a technology are largely determined by the intended purpose and the specific context in which the technology is employed (Partala & Saari, 2015). That is, the goal for which the technology is used significantly shapes users’ perceived value of the technology. Similarly, the method of use is another crucial factor influencing users’ willingness to adopt. For example, studies have shown that different methods of use can affect the extent of users’ dependence on and frequency of interaction with a technology (Hennington et al., 2009). Therefore, both the purpose and method of using GenAI can influence pre-service teachers’ user experience and behavioral habits, thereby shaping their intention to adopt GenAI in instructional design.

According to the interview results, even when teacher educators explicitly require pre-service teachers to use GenAI for instructional design, variations in their purposes and methods of use still significantly influence their willingness to adopt the technology. For example, when pre-service teachers can clearly identify the pain points in instructional design and adopt appropriate strategies to effectively address them, the performance gains brought by GenAI become more apparent. In contrast, if the purpose of use is vague or the methods are inappropriate, the value of GenAI may not be fully realized and could even lead to negative outcomes. This suggests that the adoption of GenAI by pre-service teachers is not solely dependent on the technology itself, but more importantly on “what GenAI is used for” and “how GenAI is used”. This finding addresses the limitations of traditional technology acceptance models (e.g., TAM), which often overlook specific usage contexts (Bryan & Zuva, 2021), and offers a more context-sensitive perspective for understanding pre-service teachers’ technology adoption intentions.

In future research, more attention should be paid to the influence of GenAI’s usage context and methods on pre-service teachers. It is necessary to provide differentiated support based on their varying purposes and methods of use. For instance, researchers and developers should clearly distinguish pre-service teachers’ motivations for using GenAI (e.g., improving work efficiency or enriching teaching resources) and accordingly optimize function design and user guidance to enhance the alignment between technology and user needs. At the same time, attention should also be paid to whether the ways in which pre-service teachers use GenAI are reasonable and effective. This can be addressed through training, demonstration cases, and reflective practices, which help to guide and regulate their use of GenAI in a pedagogically sound manner (MIT Sloan Teaching & Learning Technologies, 2025).

### 5.5. Interpreting Psychological Factor

This study demonstrates that the psychological factor consists of three key components: trust, perceived risk, and professional self-concept. These dimensions collectively influence pre-service teachers’ psychological acceptance of GenAI. The findings highlight the critical role of trust in shaping usage intention, while also confirming that perceived risk is a major barrier to the adoption of new technologies (Kenesei et al., 2025). When GenAI is able to build pre-service teachers’ trust and effectively reduce their perceived risks, their willingness to adopt the technology increases. Conversely, a lack of trust or heightened risk perception among pre-service teachers may hinder their willingness to adopt the technology. Therefore, building trust and mitigating perceived risks are essential preconditions for the effective implementation of GenAI in education (Sidhpurwala et al., 2025). In addition, this study also addresses the role of professional self-concept. Prior research suggests that individuals who are eager to improve their professional skills and who maintain an open attitude toward new technologies are more likely to trust and experiment with emerging tools.

According to the interview results, in the process of using GenAI to assist instructional design, trust significantly reduces pre-service teachers’ concerns about potential risks associated with GenAI, thereby removing barriers and facilitating their willingness to adopt the technology. Conversely, in the absence of trust, even the most appealing features of GenAI may fail to convince pre-service teachers if their perceived risk remains high. This finding confirms previous research suggesting that trust functions as a mediating factor in the technology acceptance process by mitigating perceived risk (Kenesei et al., 2025).

In light of the significant role of psychological factors, future research should place greater emphasis on fostering pre-service teachers’ positive psychological orientation toward GenAI. First, efforts should be made to enhance pre-service teachers’ sense of trust in GenAI. For example, training sessions can be implemented to help them understand how GenAI works, thereby increasing the transparency of its decision-making processes. Second, it is essential to reduce pre-service teachers’ perceived risks regarding GenAI. This can be achieved by offering adequate technical support and ensuring that they understand that using GenAI will not result in unacceptable or uncontrollable negative consequences. Finally, teacher education institutions should prioritize the development of pre-service teachers’ professional self-concept. This may include training and knowledge-sharing activities designed to encourage a sense of technological sensitivity and openness toward GenAI. In sum, only by simultaneously fostering trust, mitigating perceived risks, and strengthening professional identity can a positive psychological disposition toward GenAI be effectively cultivated among pre-service teachers.

### 5.6. Understanding the Interrelationships Among Four Influencing Factors

This study, through the grounded theory approach, constructs a four-dimensional model consisting of “Technical Factors– Environmental Factors– Features of Use– Psychological Factors.” This model not only identifies the key elements influencing pre-service teachers’ intention to use GenAI-assisted instructional design, but it also reveals the dynamic interrelationships among these factors. In contrast to traditional approaches that conceptualize technology adoption intention as a linear outcome driven by a single variable, this study emphasizes that pre-service teachers’ usage intention is shaped by the interactions among technical factors, environmental conditions, usage features, and psychological attributes. These four categories are not isolated but interact continuously within specific instructional contexts, mutually reinforcing or constraining one another. This dynamic mechanism aligns closely with Bandura’s triadic reciprocal determinism, which highlights the reciprocal interplay among personal factors, behavior, and environmental influences (Bandura, 2006). Moreover, this study extends classical technology acceptance models by incorporating the dimensions of features of use and psychological factors, thereby enhancing the model’s contextual relevance and explanatory power. An important innovation of this study lies in its integration of theoretical constructs derived from quantitative research with the interpretive depth of qualitative analysis. On the one hand, we draw on established constructs from quantitative models—such as relative advantage and ease of use—which provide a solid theoretical foundation for model development. On the other hand, the qualitative analysis retains openness and contextual richness, enabling more nuanced interpretations of these variables. Consequently, the model proposed in this study not only responds to classic concerns in technology acceptance research but also offers a theoretically grounded and context-sensitive framework for understanding the factors shaping pre-service teachers’ intentions to use GenAI in instructional design.

## 6. Conclusions

This paper analyzes and explores the factors influencing pre-service teachers’ usage intention to use GenAI-assisted instructional design using a grounded theory qualitative research paradigm. This study demonstrated that different pre-service teachers’ usage intention to use GenAI-assisted instructional design can be categorized into three types: positive, neutral, and negative use based on the degree of subjective use. In addition, this study identified four interrelated dimensions of influence: technical factors (relative advantage and ease of use), environmental factors (social influence, opinion leaders, and facilitating conditions), usage characteristics (purpose of use and method of use), and psychological factors (trust, perceived risk, and professional self-concept). The findings further reveal that pre-service teachers’ willingness to apply GenAI in instructional design is not determined by any single factor, but rather results from the combined influence of these four categories. Pre-service teachers use GenAI to create scenarios, design real-life examples, and obtain resource support to enhance the connection between course content and the real world. Therefore, in the field of teacher education, differentiated guidance strategies should be developed for pre-service teachers with different types of usage intention. Specifically, for pre-service teachers with active usage intentions, advanced training can be provided to explore the innovative applications of GenAI in instructional design; for those with neutral intentions, successful case-sharing sessions and hands-on workshops should be organized to strengthen their knowledge of the potential benefits of the technology; and for those with passive usage intentions, the root causes of their resistance should be analyzed in depth, and their concerns should be addressed through one-on-one communication and personalized guidance. This study contributes to the theoretical advancement of technology acceptance research by extending the conventional linear models toward a more ecological and interactive framework. On the practical level, it offers actionable recommendations for teacher education universities and institutions regarding the application of GenAI. Specifically, teacher education curricula should incorporate specialized training in GenAI technologies, enhance technical support systems, and attend to pre-service teachers’ emotional needs and professional identity formation, so as to improve their acceptance of and willingness to use GenAI tools.

Although this study has made some progress, there are still shortcomings. First, although the adoption of grounded theory can better explain pre-service teachers’ usage intention to use GenAI to assist instructional design, it will be affected by subjective cognition and pre-existing experience, and the coding process will be affected by subjectivity and lack of rigor (Timonen et al., 2018). Secondly, the total sample size of this study is small and does not cover a sufficiently diverse range of disciplines; future research should appropriately expand the sample size and try to cover all disciplines so as to improve the accuracy of the findings. Finally, the data in this study were mainly derived from qualitative sources. Although this method is helpful for deeply exploring the underlying motivations behind pre-service teachers’ usage intention to adopt GenAI-assisted instructional design, it still has certain limitations. Future research could incorporate multiple data sources for triangulation. For example, questionnaire surveys could be used to obtain a larger and more diverse sample across various application contexts, or process data could be collected and tracked to capture pre-service teachers’ actual use of GenAI, thereby enhancing the breadth and explanatory power of the findings. In order to further investigate the relationships among different influencing factors, future studies may also consider adopting fuzzy-set Qualitative Comparative Analysis (FsQCA) to examine whether multiple causal pathways or configurations exist in shaping pre-service teachers’ usage intentions. FsQCA is a method that integrates the strengths of both qualitative and quantitative approaches, emphasizing the combinatorial effects of different conditions and the causal complexity among them. It is particularly suitable for exploring how “multiple factors jointly influence the outcome variable in different ways” (Pappas & Woodside, 2021). By applying this approach, researchers could further test and deepen the understanding of the multi-path mechanisms influencing pre-service teachers’ technology adoption intentions.

## Figures and Tables

**Table 1:** Questions in the interview form.

No.	Relationship Structure
1	Why did you initially choose to use GenAI to aid in instructional design?
2	What are the biggest advantages and disadvantages over traditional instructional design?
3	What factors would motivate you to use GenAI to aid in instructional design?
4	What concerns do you have about GenAI-assisted instructional design?
5	What do you think about the future of GenAI-assisted instructional design?
6	Will you continue to use GenAI-assisted instructional design as a future teacher?

**Table 2:** Interviewee information sheet.

Serial Number	Gender	Educational Background	Specialty	Internships and Teaching Periods
A	Female	Undergraduate	Educational Technology	Primary School
B	Female	Undergraduate	Primary Education	Primary School
C	Male	Undergraduate	Educational Technology	Primary School
D	Female	Postgraduate	Modern Educational Technology	Primary School
E	Female	Postgraduate	Primary Education	Primary School
F	Female	Undergraduate	Educational Technology	Primary School
G	Female	Postgraduate	Educational Technology	Middle School
H	Male	Postgraduate	Subject-specific Mathematics	Primary School
I	Female	Undergraduate	Language and Literature	High School
J	Male	Undergraduate	Language and Literature	Middle School
K	Female	Postgraduate	English	High School
L	Female	Undergraduate	English	Middle School
M	Male	Postgraduate	Modern Educational Technology	Middle School
N	Female	Undergraduate	English	Primary School
O	Female	Postgraduate	Modern Educational Technology	Primary School
P	Female	Postgraduate	Language and Literature	Middle School
Q	Female	Undergraduate	Educational Technology	Middle School
R	Male	Postgraduate	Modern Educational Technology	Middle School
S	Male	Undergraduate	Subject-specific Mathematics	High School
T	Female	Postgraduate	Language and Literature	Middle School
U	Female	Undergraduate	Language and Literature	Middle School
V	Male	Postgraduate	Educational Technology	Primary School
W	Female	Postgraduate	Modern Educational Technology	Primary School

**Table 3:** Selective coding.

Typical Relationships	Relationship Structure	Connotation
Technical Factor —Usage Intention	Causal Relationship	GenAI’s usefulness and application affect pre-service teachers’ usage intention.
Environmental Factor —Usage Intention	Causal Relationship	Social influence, opinion leaders, and external support can influence pre-service teachers’ usage intention.
Features of Use —Usage Intention	Causal Relationship	Pre-service teachers’ purpose of use and how they use it can affect their usage intention.
Emotional Factor —Usage Intention	Causal Relationship	Pre-service teachers’ trust, perceived risk, and professional emotional connections to GenAI affect their usage intention.

## Data Availability

The data reported in this study are available upon request from the corresponding authors.

## Appendix A. Open Coding

**Original Statement**	**Initial Concepts**	**Initial Category**
The use of this tool contributed to time savings and enhanced the overall efficiency of instructional planning.	Increase Efficiency	Relative Advantage
Its use facilitates the retrieval of high-quality resources and exemplary cases, which in turn contributes to the improvement of instructional design quality.	Enhancing Quality	
It offers a wide variety of instructional resources, supporting teachers in developing more flexible and context-responsive lesson plans.	Resource-rich	
It assisted in designing clear learning objectives and breaking them down into manageable sub-tasks. As students completed each task, they felt a sense of progress toward the overall goal, which in turn enhanced their engagement and interest in learning.	Student Interest Stimulation	
It offers innovative instructional ideas and facilitates the design of engaging learning activities.	Innovation Inspiration	
It provides innovative instructional ideas and supports the design of engaging learning activities.	Personalized Support	
The tool features a user-friendly interface and is easy to use.	Easy Operation	Ease of Use
Using GenAI requires relatively little time and effort to learn.	Low Learning Costs	
Seeing my classmates use GenAI motivated me to give it a try.	Peer Influence	Social Influence
Using GenAI is perceived as a form of innovation and progress.	Social Recognition	
Information push from social media platforms serves as a catalyst for increasing my awareness of and engagement with GenAI in instructional design.	Online Promotion	
My mentor teacher or university instructors recommended the use of GenAI.	Mentor Recommendation	Opinion Leader
It is widely believed in the field of education that using GenAI to support teaching and learning is a future trend.	Industry Trend	
The institution offers access to GenAI tools and supporting resources to facilitate instructional design and teaching practices.	Resource Support	Facilitating Conditions
The institution provides training programs on the use of GenAI to support instructional design and teaching.	Training Support	
There are institutional or governmental policies in place to support the application of GenAI in education.	Policy Support	
I often use GenAI to generate instructional materials, such as slide content and visual resources.	Courseware Generation	Purpose of Use
I use GenAI to check and polish the textual content of instructional materials to ensure clarity and accuracy.	Language Editing and Proofreading	
Different prompts can train GenAI to generate different types of content.	Question Formulation	Method of Use
The content generated by GenAI requires users to selectively extract relevant information, as it cannot be adopted in its entirety without critical judgment.	Screening Method	
DouBao’s advanced language generation helps me efficiently create lesson plans and instructional activities.	GenAI Type	
It effectively retrieves high-quality resources, including subject materials and interest-based cases, making it my primary tool for sourcing teaching content.	High Trust	Trust
Though the interactive formats suggested by GenAI seemed attractive, they failed to engage students effectively and did not achieve the expected results.	Lack of Trust	
I am concerned about the potential inaccuracy or lack of reliability in the content produced by GenAI.	Technophobia	Perceived Risk
I am concerned that using GenAI might involve copyright infringement or violations of personal privacy.	Ethical Concerns	
While GenAI can efficiently generate teaching content, it occasionally produces repetitive or uninspired outputs and may include occasional inaccuracies.	Content quality	
Although the instructional designs generated by GenAI are well-structured, I feel that my creativity is being constrained. For example, most of the classroom activities are provided by GenAI, leaving me with few opportunities to contribute my own ideas.	Individual creativity	
With the availability of GenAI-generated teaching materials, I’ve noticed a decline in my deep understanding of subject knowledge. As I increasingly rely on GenAI, I worry that my professional competence may gradually deteriorate.	De-skilling of professional competence	
After using GenAI to assist with instructional design, I felt a diminished sense of professional identity. Many tasks that I used to perform were replaced by GenAI, making me question my value as a teacher.	Sense of Professional Identity	Professional Self-Concept
After using GenAI for instructional support, I often felt like a mere “content handler” since much of the material was directly generated by the tool. The sense of value I used to derive from deep engagement in the design process has gradually diminished.	Sense of Professional Value	
GenAI has significantly reduced the time I spend on lesson preparation while enhancing overall teaching effectiveness. Now, whenever I need to design instruction, turning to GenAI has become my first instinct.	Active Usage Intention	Usage Intention
The instructional designs generated by GenAI are overly standardized and do not align with my personal teaching style. Instead of assisting me, it disrupts my instructional flow.	Negative Usage Intention	
While GenAI offers certain advantages in instructional design—such as convenient access to relevant resources—it also has limitations. For instance, the generated content is not always well-aligned with real classroom contexts. Therefore, I decide whether to use it based on the specific needs of each teaching situation.	Neutral Usage Intention	

## Appendix B. Axial Coding

**Major Category**	**Initial Category**	**Category Connotation**
Technical Factor	Relative Advantage	The practical value and positive effect of GenAI in enhancing various aspects of pre-service teachers’ instructional design.
	Ease of use	The fact that GenAI provides a simple and intuitive interactive interface, allowing pre-service teachers to get started quickly without complicated operation steps.
Environmental Factor	Social Influence	The influence of social factors on pre-service teachers’ use of GenAI to support instructional design.
	Opinion Leader	The influence of a group of people with a certain level of prestige and social status on pre-service teachers’ use of GenAI-assisted instructional design.
	Facilitating Conditions	These components work synergistically to construct a supportive ecosystem that empowers pre-service teachers to engage in effective GenAI-assisted instructional design.
Usage characteristics	Purpose of Use	The functional goals that preservice teachers aim to achieve through the use of GenAI tools, which are directly aligned with the specific task demands of instructional design.
	Method of Use	How GenAI is employed to support in instructional design.
Psychological Factor	Trust	The level of trust and recognition of GenAI-assisted instructional design by pre-service teachers.
	Perceived Risk	Pre-service teachers’ subjective perceptions of the potential risks associated with using GenAI-assisted instructional design.
	Professional Self-Concept	The impact of GenAI-assisted instructional design on pre-service teachers’ professional identity and sense of professional value.
Usage Intention	Active Usage Intention	GenAI-assisted instructional design is more recognized and usage intention is more positive.
	Negative Usage Intention	Disapproval of GenAI-assisted instructional design and more negative usage intention.
	Neutral Usage Intention	Attitudes towards GenAI-assisted instructional design are unclear and usage intention is not evident.
